# Risk of Pleural Empyema in Adult Patients With Asthma: A Nationwide Retrospective Cohort Study

**DOI:** 10.3389/fmed.2022.851573

**Published:** 2022-04-04

**Authors:** Wei-Chih Liao, Cheng-Li Lin, Te-Chun Shen, Chih-Yen Tu, Te-Chun Hsia, Wu-Huei Hsu

**Affiliations:** ^1^Division of Pulmonary and Critical Care Medicine, Department of Internal Medicine, China Medical University Hospital, Taichung, Taiwan; ^2^School of Medicine, China Medical University, Taichung, Taiwan; ^3^Management Office for Health Data, China Medical University Hospital, Taichung, Taiwan; ^4^Intensive Care Unit, Chu Shang Show Chwan Hospital, Nantou, Taiwan

**Keywords:** empyema, asthma, pneumonia, cohort study, retrospective study

## Abstract

**Background:**

Respiratory system infections commonly occur among individuals with asthma. However, whether asthma patients have a higher risk of pleural empyema development remains unclear.

**Methods:**

This is a retrospective cohort study based on data from the National Health Insurance Research Database of Taiwan. The asthma cohort consisted of 48,360 newly diagnosed adult individuals from 2000 to 2012. The comparison cohort consisted of the same number of adults who did not have asthma and was matched for age, gender, comorbidity, and the year of diagnosis. The development of pleural empyema was followed up to 2013.

**Results:**

Pleural empyema incidence was 2.03-fold higher in the asthma cohort compared to the comparison cohort (8.65 vs. 4.25 per 10,000 person-years), with an adjusted hazard ratio (HR) of 2.12 [95% confidence interval (CI) = 1.76–2.56]. Stratified analyses by age, gender, comorbidity, and corticosteroid use revealed that the crude and adjusted HRs of pleural empyema associated with asthma were all significant. Among patients with asthma, the risk of pleural empyema elevated with increased frequency of annual asthma-related emergency room visits and hospital admissions (≥1 vs. <1, aHR = 8.07, 95% CI = 4.31–15.1 and aHR = 9.31, 95% CI = 5.56–15.6).

**Conclusion:**

An increased risk of pleural empyema occurrence was observed in adult patients with asthma than those without asthma. Furthermore, the risk of pleural empyema may increase with poor control of asthma.

## Introduction

Asthma is a heterogeneous disease manifesting with airway inflammation (1). This disease is defined by the presence of respiratory symptoms that vary over time and in intensity, together with variable expiratory airflow limitation (2). Inadequate control of asthma may lead to frequent exacerbations, worse health status, and poor quality of life (3). Asthma is also found to be associated with respiratory system infections, but information on the underlying mechanism of this predisposition is limited (4). Altered epithelial microenvironment and impaired immune function may contribute to the susceptibility of respiratory system infections (5, 6).

Pleural empyema indicates the occurrence of frank pus within the pleural space (7). It is most commonly caused by respiratory system infections, such as pneumonia (8). Mortality rate among individuals having pneumonia with pleural empyema is far higher than in patients who do not have pleural empyema (9, 10). The crucial nature of pleural empyema and the need for removal have been identified for several centuries (11). Delayed diagnosis and poor drainage have been found to be linked with high mortality (12). Alcohol consumption, substance use, diabetes mellitus, immunosuppression, malignancy, pulmonary disease, and prior occurrence of pleural effusion are predictors of the development of pleural empyema (13, 14).

Several studies have investigated the association between asthma and respiratory system infections (15–18). However, the studies did not examine the incidence of pleural empyema in asthma patients. Pleural empyema is a noticeable infection of the respiratory system and requires timely treatments, such as antibiotic therapy, pleural drainage, intrapleural fibrinolysis, and surgery. Thus, investigating the risk of pleural empyema in patients with asthma is necessary. This study aimed to examine whether patients with asthma have a higher risk of pleural empyema development. In addition, we attempted to assess the effect of asthma control on the occurrence of pleural empyema.

## Materials and Methods

### Data Source

The Taiwan National Health Insurance (NHI) program was established in 1995. The National Health Insurance Research Database (NHIRD) is a nationwide database housing medical claims data of over 99.5% of people living in Taiwan (https://nhird.nhri.org.tw/en/). The database is updated and maintained by the National Health Research Institutes. The Longitudinal Health Insurance Database 2000 (LHID2000, a subset data of NHIRD) was used for this study. The database contains medical claims data of one million persons randomly selected from users registered in 2000. Data on their demographic characteristics, diagnostic codes, procedure claims, and medication claims were available from 1995 to 2013. All data were deidentified to protect their privacy; therefore, written informed consent from the participants involved was unnecessary. This study was approved by the Research Ethics Committee of the China Medical University and Hospital (CMUH-104-REC2-115).

### Study Cohorts

Patients with newly diagnosed asthma [International Classification of Disease, 9th Revision, Clinical Modification (ICD-9-CM) code 493 and asthma medication (bronchodilator or corticosteroid)] from January 1, 2000, to December 31, 2012, were selected in the asthma cohort. The date of the diagnosis was defined as the index date. Individuals with a diagnosis of pleural empyema before the index date and those with incomplete data were excluded from the analysis. The individuals in the comparison cohort included people free from asthma. The exclusion criteria for the comparison cohort were the same as for the asthma cohort. The comparison cohort was 1:1 frequency-matched with the asthma cohort by age, gender, comorbidity, and index year. All subjects were monitored until any of the following occurred: (1) development of pleural empyema, (2) withdrawal from NHI system, (3) death, and (4) the date of 31st of December 2013.

### Outcome and Variables

All diseases were recorded in accordance with the ICD-9-CM in the NHIRD. The primary outcome was pleural empyema (based on ICD-9-CM code 510 and related antibiotic treatment). We also determined the related baseline comorbidities between 1995 and index date, including chronic obstructive pulmonary disease (COPD, ICD-9-CM code 496), diabetes mellitus (ICD-9-CM code 250), chronic kidney disease (CKD, ICD-9-CM code 585), chronic liver disease and cirrhosis (CLD, ICD-9-CM code 571), rheumatic disease (ICD-9-CM codes 446.5, 710.0–710.4, 714.0–714.2, 714.8, and 725), stroke (ICD-9-CM codes 433–438), cancer (ICD-9-CM codes 140–209), and malnutrition (ICD-9-CM codes 260–269). We selected only diagnoses from the outpatient department that appeared at least twice within 1 year or had a diagnosis of hospitalization to increase the accuracy for asthma, pleural empyema, and all comorbidities. In addition, we evaluated the related medication, corticosteroid use.

### Statistical Analysis

We used the Chi-squared test to examine the proportion distribution of age group, gender, comorbidity, and medication between asthma and comparison cohorts. The means of age in the two cohorts were compared using a student's *t*-test. The estimation of cumulative incidence of pleural empyema in asthma and comparison cohorts was performed by the Kaplan–Meier method. A log-rank test was utilized to determine the significance. The incidence rates of pleural empyema were calculated by asthma, age group, gender, comorbidities, and medication. Univariable and multivariable Cox proportional hazard regression models were used to estimate hazard ratios (HRs) and 95% confidence intervals (CIs). Moreover, we calculated the incidence rates and relative risk of pleural empyema by stratification with age, gender, comorbidities, and medication between asthma and comparison cohorts. Among patients with asthma, we further evaluated the impact of the annual number of asthma-related emergency room visits and hospital admissions and cumulative corticosteroid doses on pleural empyema development. Data analysis was performed with the SAS statistical software (Version 9.4 for Windows; SAS Institute, Inc., Cary, NC, USA). Statistical significance was considered at a *p*-value < 0.05.

## Results

**Figure 1:**
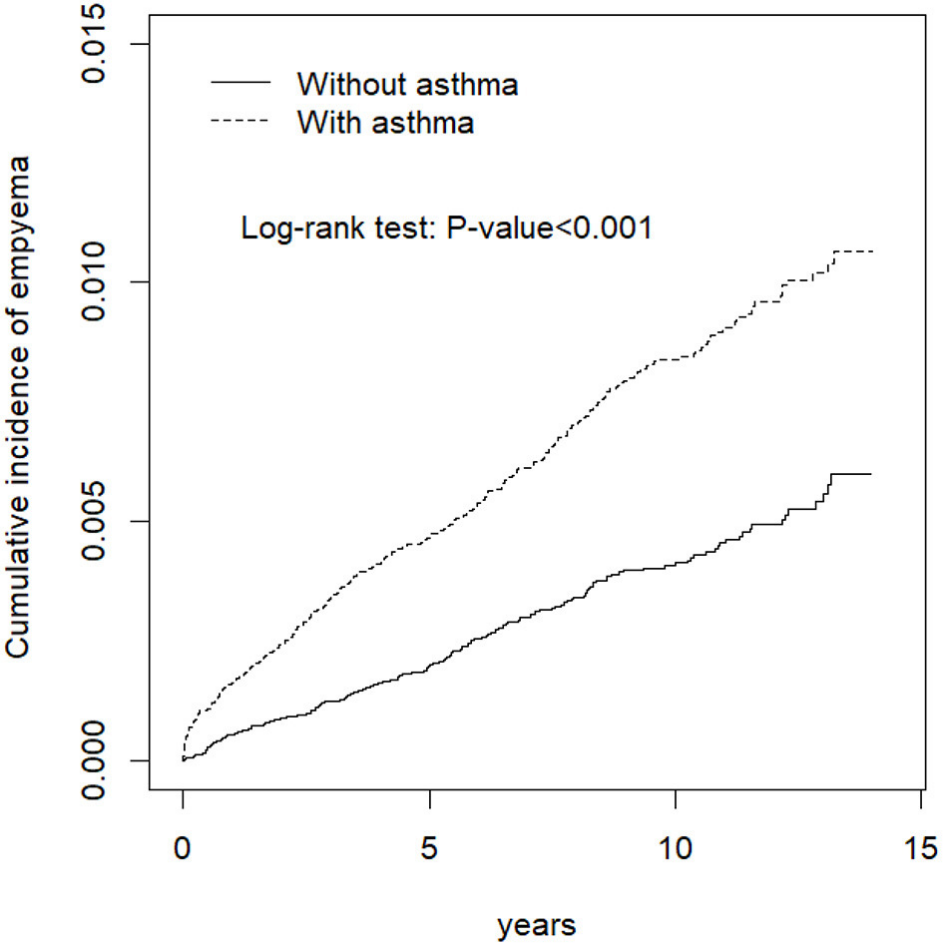
Cumulative incidence of pleural empyema in the asthma group (dashed line) and in the comparison group (solid line).

We recruited an asthma group comprising 48,360 patients and a comparator group of 48,360 individuals (Table 1). Age and gender did not significantly differ between asthma and comparator group. The mean age ± standard division of asthma and comparator group was 54.9 ± 18.7 and 54.1 ± 18.5 years, respectively. Approximately 52% of the individuals were women in both groups. The major comorbidities of the asthma group were COPD (24.8%), CLD (21.7%), followed by diabetes mellitus (10.9%), stroke (6.93%), and cancer (3.16%). The mean follow-up periods were 7.72 ± 4.15 years in the asthma group and 8.03 ± 4.02 years in the comparator group. The proportion of corticosteroid use were 23.6% in the asthma group and 15.2% in the comparator group. Figure 1 showed that patients with asthma had a higher cumulative incidence of pleural empyema than individuals without asthma throughout the 14-year study period.

**Table 1:** Baseline characteristics between asthma and non-asthma cohorts.

	**Asthma**				***p*-value**
	**No**		**Yes**		
	***N*** **=** **48,360**		***N*** **=** **48,360**		
	***n***	**%**	***n***	**%**	
**Age**					0.63
20–49	19,720	40.8	19,680	40.7	
50–64	11,918	24.6	11,826	24.5	
≥65	16,722	34.6	16,854	34.9	
Mean ± SD	54.1	±18.5	54.9	±18.7	0.001
**Gender**					0.46
Women	24,873	51.4	24,988	51.7	
Men	23,487	48.6	23,372	48.3	
**Comorbidity**					
Diabetes mellitus	5,254	10.9	5,269	10.9	0.88
CKD	1,195	2.47	1,066	2.20	0.01
CLD	10,337	21.4	10,513	21.7	0.17
COPD	11,880	24.6	11,988	24.8	0.42
Rheumatic disease	1,360	2.81	1,346	2.78	0.78
Stroke	3,549	7.34	3,350	6.93	0.01
Cancer	1,598	3.30	1,527	3.16	0.20
Malnutrition	401	0.83	339	0.70	0.02
**Medication**					
Corticosteroid	7,355	15.2	11,392	23.6	<0.001

*CKD, chronic kidney disease; CLD, chronic liver disease and cirrhosis; COPD, chronic obstructive pulmonary disease; SD, standard deviation*.

The overall incidence density rates of pleural empyema were 8.65 and 4.25 per 10,000 person-years in asthma and comparator groups, respectively (Table 2). Compared with the comparator group, the corresponding adjusted HR (aHR) of pleural empyema was 2.12 (95% CI = 1.76–2.56) in the asthma group after adjusting for age, gender, COPD, diabetes mellitus, CKD, CLD, stroke, cancer, and corticosteroid use. Compared with persons aged 20–49, the aHRs of pleural empyema were 2.43-fold higher in those aged 50–64 (95% CI = 1.80–3.29) and 4.10-fold higher in those aged ≥65 (95% CI = 3.09–5.44). The aHR of pleural empyema was 2.55-fold higher for men relative to women (95% CI = 2.09–3.12). Moreover, the risk of pleural empyema was higher in persons with stroke (aHR = 2.68, 95% CI = 2.12–3.38), diabetes mellitus (aHR = 1.86, 95% CI = 1.50–2.31), and COPD (aHR = 1.48, 95% CI = 1.22–1.79) compared with subjects without these comorbidities.

**Table 2:** Incidences and hazard ratios of pleural empyema by asthma, age, gender, comorbidity, and corticosteroid use among all participants.

	**Event**	**PY**	**Rate^#^**	**Crude HR (95% CI)**	**Adjusted HR^**†**^ (95% CI)**
**Asthma**					
No	165	388,334	4.25	1.00	1.00
Yes	323	373,337	8.65	2.03 (1.68–2.45)^***^	2.12 (1.76–2.56)^***^
**Age**					
20–49	69	347,688	1.98	1.00	1.00
50–64	121	196,168	6.17	3.10 (2.31–4.16)^***^	2.43 (1.80–3.29)^***^
≥65	298	217,815	13.7	6.76 (5.20–8.79)^***^	4.10 (3.09–5.44)^***^
**Gender**					
Women	135	404,727	3.34	1.00	1.00
Men	353	356,945	9.89	2.95 (2.42–3.60)^***^	2.55 (2.09–3.12)^***^
**Comorbidity**					
COPD					
No	278	569,143	4.66	1.00	1.00
Yes	210	165,529	12.7	2.68 (2.24–3.21)^***^	1.48 (1.22–1.79)^***^
Diabetes mellitus					
No	372	694,873	5.35	1.00	1.00
Yes	116	66,799	17.4	3.17 (2.57–3.91)^***^	1.86 (1.50–2.31)^***^
CKD					
No	470	749,761	6.27	1.00	1.00
Yes	18	11,911	15.1	2.30 (1.43–3.68)^***^	1.22 (0.75–1.96)
CLD					
No	350	599,220	5.84	1.00	1.00
Yes	138	162,451	8.49	1.45 (1.19–1.77)^***^	1.16 (0.95–1.41)
Rheumatic disease					
No	471	743,383	6.34	1.00	
Yes	17	18,288	9.30	1.44 (0.89–2.33)	
Stroke					
No	390	727,905	5.36	1.00	1.00
Yes	98	33,766	29.0	5.19 (4.15–6.49)^***^	2.68 (2.12–3.38)^***^
Cancer					
No	466	746,246	6.24	1.00	1.00
Yes	22	15,425	14.3	2.17 (1.41–3.33)^***^	1.39 (0.91–2.14)
Malnutrition					
No	484	756,482	6.40	1.00	
Yes	4	5,189	7.71	1.19 (0.45–3.18)	
**Medication**					
Corticosteroid					
No	370	634,551	5.83	1.00	1.00
Yes	118	127,120	9.28	1.56 (1.27–1.92)^***^	1.02 (0.82–1.26)

*CI, confidence interval; CKD, chronic kidney disease; CLD, chronic liver disease and cirrhosis; COPD, chronic obstructive pulmonary disease; HR, hazard ratio; PY, person-years*.

# *Incidence rate per 10,000 person-years*.

*Multivariable analysis including age, gender, COPD, diabetes mellitus, CKD, CLD, stroke, cancer, and corticosteroid use*.

*** *p < 0.001*.

Further analyses revealed that the incidences and aHRs of pleural empyema for the asthma group were all significantly higher compared with the comparator group after stratification for age, gender, presence of comorbidities, and corticosteroid use (Table 3). In addition, we assessed the impact of the frequency of asthma-related emergency room visits and hospital admissions on the development of pleural empyema among the asthma group (Table 4). A higher frequency of annual asthma-related emergency room visits and hospital admissions raised the risk of pleural empyema development (both *p* for trend <0.001). Moreover, we analyzed cumulative corticosteroid doses on pleural empyema development among the asthma group (Table 4). The incidence of pleural empyema was higher in those with corticosteroid use; however, the *p* value for trend was not significant (*p* = 0.25).

**Table 3:** Incidences and hazard ratios of pleural empyema by age, gender, comorbidity, and corticosteroid use between asthma and non-asthma cohorts.

	**Asthma**						**Crude HR (95% CI)**	**Adjusted HR^†^ (95% CI)**
	**No**			**Yes**				
	**Event**	**PY**	**Rate^#^**	**Event**	**PY**	**Rate^#^**		
**Age**								
20–49	23	174,025	1.32	46	173,662	2.65	2.01 (1.22–3.31)^**^	1.98 (1.20–3.27)^**^
50–64	31	99,658	3.11	90	96,510	9.33	3.00 (1.99–4.51)^***^	3.06 (2.03–4.62)^***^
≥65	111	114,651	9.68	187	103,164	18.1	1.86 (1.47–2.36)^***^	1.91 (1.51–2.43)^***^
**Gender**								
Women	36	205,289	1.75	99	199,437	4.96	2.83 (1.93–4.14)^***^	2.78 (1.89–4.08)^***^
Men	129	183,045	7.05	224	173,900	12.9	1.82 (1.47–2.26)^***^	1.94 (1.56–2.42)^***^
**Comorbidity^§^**								
No	34	212,837	1.60	98	210,361	4.66	2.92 (1.98–4.31)^***^	2.95 (1.99–4.36)^***^
Yes	131	175,497	7.46	225	162,975	13.8	1.84 (1.48–2.28)^***^	1.91 (1.54–2.37)^***^
**Corticosteroid**								
No	139	338,472	4.11	231	296,079	7.80	1.90 (1.54–2.34)^***^	2.05 (1.66–2.53)^***^
Yes	26	49,862	5.21	92	77,258	11.9	2.28 (1.48–3.53)^***^	2.43 (1.61–3.86)^***^

*CI, confidence interval; HR, hazard ratio; PY, person-years*.

# *Incidence rate per 10,000 person-years*.

† *Multivariable analysis including age, gender, COPD, diabetes mellitus, CKD, CLD, stroke, cancer, and corticosteroid use*.

§ *Individuals with any comorbidity of COPD, diabetes mellitus, CKD, CLD, rheumatic disease, stroke, cancer, and malnutrition were classified into the comorbidity group*.

** *p < 0.01*,

*** *p < 0.001*.

**Table 4:** Incidences and hazard ratios of pleural empyema by emergency room visits, hospital admissions, and cumulative corticosteroid doses among asthma cohort.

	**Event**	**Incidence^#^**	**Crude HR (95% CI)**	**Adjusted HR^†^ (95% CI)**
**Annual emergency room visits**				
<1	311	8.34	1.00	1.00
≥1	12	334.9	19.7 (10.6–36.4)^***^	8.07 (4.31–15.1)^***^
*p* for trend				<0.001
**Annual hospital admissions**				
<1	302	8.10	1.00	1.00
≥1	21	362.7	24.6 (15.0–40.5)^***^	9.31 (5.56–15.6)^***^
*p* for trend				<0.001
**Cumulative corticosteroid doses (mg)**				
None	231	7.80	1.00	1.00
<115	9	10.3	1.31 (0.67–2.55)	0.98 (0.50–1.91)
115–335	35	15.4	1.93 (1.35–2.75)^***^	1.53 (1.07–2.19)^*^
≥335	48	10.6	1.30 (0.95–1.78)	0.93 (0.68–1.28)
*p* for trend				0.25

*CI, confidence interval; HR, hazard ratio*.

# *Incidence rate per 10,000 person-years*.

† *Multivariable analysis including age, gender, COPD, diabetes mellitus, CKD, CLD, stroke, cancer, and corticosteroid use*.

* *p < 0.05*,

*** *p < 0.001*.

## Discussion

We believed that this investigation is the first population-based retrospective cohort study to evaluate the incidence of pleural empyema in patients with asthma. Our findings revealed that patients with asthma have a significantly higher risk of developing pleural empyema than those without asthma. The risk of pleural empyema was also larger in older people, in males, those with comorbidities. Furthermore, the hazards of pleural empyema were significantly larger in the asthma cohort compared with the comparison cohort under stratification by age, gender, comorbidity, and corticosteroid use. Moreover, we found that the risk of pleural empyema was higher in asthma patients with an increased number of asthma-related emergency medical demands and hospital admissions, indicating that the level of asthma control may influence the occurrence of pleural empyema.

The mechanism between asthma and pleural empyema remains largely unknown. Patients with asthma who are susceptible to pneumonia may still play a major role. This condition may be driven by the following: (1) a large prevalence of carriage of the bacteria, (2) a disordered immune response from exposure to the bacteria, (3) impaired bacterial clearance, and (4) a suboptimal response to vaccination (5). Parapneumonic pleural effusions are known to represent a common complication of pneumonia and can be found in approximately 40% of bacterial pneumonia cases (11). In addition, inhaled or systemic corticosteroid use, shared comorbidities, cigarette smoking, alcohol consumption, unhealthy lifestyle, poor self-care, and poor physical health are commonly noted among asthma patients. These factors are also related to the development of pneumonia and pleural empyema (9).

Inconsistent evidence was found between chronic inflammatory airway disease and the development of pleural empyema. Lu et al. (19) conducted a case-control study to evaluate the potential risk factors of pleural empyema. They enrolled 1,851 pleural empyema cases and 7,404 non-empyema controls and found significant factors that lead to pleural empyema include the following: aspiration history [odds ratio (OR) = 7.28, 95% CI = 5.00–10.6)], human immunodeficiency virus infection (OR = 5.66, 95% CI = 1.38–23.2), malnutrition (OR = 2.86, 95% CI = 2.07–3.95), cancer (OR = 2.74, 95% CI = 2.28–3.30), diabetes mellitus (OR = 2.25, 95% CI = 1.96–2.59), stroke (OR = 1.99, 95% CI = 1.70–2.34), CKD (OR = 1.78, 95% CI = 1.42–2.25), chronic obstructive pulmonary disease (COPD, OR = 1.72, 95% CI = 1.47–2.01), asthma (OR = 1.34, 95% CI = 1.15–1.57), and CLD (OR = 1.20, 95% CI = 1.06–1.35). In another study, Lu et al. evaluated COPD and the subsequent development of pleural empyema (20). They enrolled 55,136 COPD cases and 98,769 non-COPD controls and found that the incidence of pleural empyema was 3.64-fold higher in the COPD cohort than in the comparison cohort (15.8 vs. 4.34 per 10,000 person-years), with a corresponding aHR of 3.25 (95% CI = 2.73–3.87). These findings may suggest that chronic inflammatory airway disease may contribute to the development of pleural empyema. By contrast, Dusemund et al. (21) performed a case-control study in Switzerland to investigate outcomes of community-acquired pneumonia in patients with chronic lung disease. They found that the incidence of pleural empyema was insignificant in asthma [0.5% (asthma) vs. 0.9% (controls), *p* = 0.141] and COPD [0.5% (COPD) vs. 0.5% (controls), *p* = 0.817]. In addition, Elemraid et al. (22) assessed predictors of pleural empyema development in children. They found that age, sex, mother's age, smoking among the child's parents, poverty, nursery attendance, asthma, and household characteristics (bedrooms and number of occupants) were not significantly related. Therefore, we may need additional investigations to clarify this issue.

### Strength

The strength of this study lies in the establishment of a population-based asthma cohort to assess the risk of developing pleural empyema. Conducting a prospective cohort study is expensive. Therefore, a retrospective cohort study based on insurance claims data may be a suitable and economical alternative. Regardless of socioeconomic background and/or residential location, the universal coverage by the NHI program lowers access barriers to health care all citizens (23). This study was able to reflect a “real world” scenario in which asthma, pleural empyema, and comorbidities were assessed during medical evaluation.

### Limitation

Several limitations exist and need to be considered in interpreting the study findings. First, the ICD-9-CM algorithm was used to define asthma, pleural empyema, and comorbidities. All diagnoses were dependent on the competence of clinical physicians in diagnosing; however, asthma has been carefully validated in the NHIRD (24). In addition, an *ad hoc* committee established by the insurance authority monitored the evaluation of claims data to prevent errors and violations. We selected only diagnoses from the outpatient department that appeared at least twice within 1 year or had a diagnosis of hospitalization to increase the accuracy. In addition, we applied the medication used to improve the diagnosis of asthma and empyema. Second, the NHIRD does not provide detailed information regarding smoking habits, drinking habits, and other environmental factors, which are potentially confounding factors in the current study. In addition, relevant clinical variables, such as serum laboratory data, image reports, and culture results, were unavailable in the database.

## Conclusion

An increased risk of pleural empyema occurrence was observed in adult patients with asthma compared to those without asthma. Furthermore, the risk of pleural empyema may increase with the degree of asthma control.

## Data Availability Statement

The original contributions presented in the study are included in the article/supplementary material, further inquiries can be directed to the corresponding author.

## Author Contributions

W-CL, T-CS, C-YT, T-CH, and W-HH: conception and design. C-YT, T-CH, and W-HH: administrative support. W-CL, C-LL, and T-CS: collection and assembly of data and data analysis and interpretation. All authors: manuscript writing and final approval of manuscript.

## Funding

This study was supported by China Medical University Hospital (DMR-110-033).

## Conflict of Interest

The authors declare that the research was conducted in the absence of any commercial or financial relationships that could be construed as a potential conflict of interest.

## Publisher's Note

All claims expressed in this article are solely those of the authors and do not necessarily represent those of their affiliated organizations, or those of the publisher, the editors and the reviewers. Any product that may be evaluated in this article, or claim that may be made by its manufacturer, is not guaranteed or endorsed by the publisher.
